# Analysis of 100,000 human cancer genomes reveals the landscape of tumor mutational burden

**DOI:** 10.1186/s13073-017-0424-2

**Published:** 2017-04-19

**Authors:** Zachary R. Chalmers, Caitlin F. Connelly, David Fabrizio, Laurie Gay, Siraj M. Ali, Riley Ennis, Alexa Schrock, Brittany Campbell, Adam Shlien, Juliann Chmielecki, Franklin Huang, Yuting He, James Sun, Uri Tabori, Mark Kennedy, Daniel S. Lieber, Steven Roels, Jared White, Geoffrey A. Otto, Jeffrey S. Ross, Levi Garraway, Vincent A. Miller, Phillip J. Stephens, Garrett M. Frampton

**Affiliations:** 1 Foundation Medicine Inc., 150 Second St., Cambridge, MA 02141 USA; 2 Dana-Farber Cancer Institute, Harvard Medical School, Boston, Massachusetts USA; 3 Broad Institute of MIT and Harvard, Cambridge, Massachusetts USA; 4 The Hospital for Sick Children, Toronto, Ontario Canada

**Keywords:** Tumor mutational burden, Cancer genomics, Mismatch repair, PMS2

## Abstract

**Background:**

High tumor mutational burden (TMB) is an emerging biomarker of sensitivity to immune checkpoint inhibitors and has been shown to be more significantly associated with response to PD-1 and PD-L1 blockade immunotherapy than PD-1 or PD-L1 expression, as measured by immunohistochemistry (IHC). The distribution of TMB and the subset of patients with high TMB has not been well characterized in the majority of cancer types.

**Methods:**

In this study, we compare TMB measured by a targeted comprehensive genomic profiling (CGP) assay to TMB measured by exome sequencing and simulate the expected variance in TMB when sequencing less than the whole exome. We then describe the distribution of TMB across a diverse cohort of 100,000 cancer cases and test for association between somatic alterations and TMB in over 100 tumor types.

**Results:**

We demonstrate that measurements of TMB from comprehensive genomic profiling are strongly reflective of measurements from whole exome sequencing and model that below 0.5 Mb the variance in measurement increases significantly. We find that a subset of patients exhibits high TMB across almost all types of cancer, including many rare tumor types, and characterize the relationship between high TMB and microsatellite instability status. We find that TMB increases significantly with age, showing a 2.4-fold difference between age 10 and age 90 years. Finally, we investigate the molecular basis of TMB and identify genes and mutations associated with TMB level. We identify a cluster of somatic mutations in the promoter of the gene *PMS2*, which occur in 10% of skin cancers and are highly associated with increased TMB.

**Conclusions:**

These results show that a CGP assay targeting ~1.1 Mb of coding genome can accurately assess TMB compared with sequencing the whole exome. Using this method, we find that many disease types have a substantial portion of patients with high TMB who might benefit from immunotherapy. Finally, we identify novel, recurrent promoter mutations in *PMS2*, which may be another example of regulatory mutations contributing to tumorigenesis.

**Electronic supplementary material:**

The online version of this article (doi:10.1186/s13073-017-0424-2) contains supplementary material, which is available to authorized users.

## Background

In recent years, immunotherapies have shown great promise as treatments for skin, bladder, lung, and kidney cancers, and also for tumors which are mismatch repair deficient, with extremely durable responses for some patients [1–6]. These agents modulate the pathways that control when and where immune responses are mounted, increasing antitumor activity through immune checkpoint blockade [7]. Inhibitors of cytotoxic T lymphocyte-associated antigen 4 (CTLA-4) [8, 9] and of programmed cell death protein 1 (PD-1) receptor [10] were the first drugs of this type, which promote T-cell activation [2]. Other agents targeting immune checkpoint pathways are now approved or in active preclinical and clinical development [11–17].

While treating cancer with immunotherapy can be highly effective, only some patients respond to these treatments [18]. Given the promise these agents have shown in treatment of refractory disease and the durable responses that occur in some cases, there is great interest in identifying patients who are most likely to derive benefit from these therapies. Assays that measure PD-1/PD-L1 protein expression by immunohistochemistry (IHC) are approved as complementary or companion diagnostics for some of these drugs; however, measurement of PD-1/PD-L1 expression is technically challenging, can be difficult to interpret, and is not always an accurate predictor of response to immunotherapy [19]. An emerging biomarker for response to immunotherapy is the total number of mutations present in a tumor specimen. This is termed the mutation load or tumor mutational burden (TMB). It is hypothesized that highly mutated tumors are more likely to harbor neoantigens which make them targets of activated immune cells. This metric has been shown, in several tumor types, to correlate with patient response to both CTLA-4 and PD-1 inhibition [4, 20, 21]. In fact, in one clinical trial, TMB was more significantly associated with response rate than expression of PD-L1 by immunohistochemistry [6]. Neoantigen load has also been correlated with response to immunotherapy [22]. However, no recurrent neoantigens have been found that predict response to date [23].

Increased mutation rate is a well-characterized feature of human cancer. Abnormal activity in several cellular pathways, including DNA damage repair and DNA replication, can increase the overall rate of somatic mutations in tumors, as can exposure to mutagens such as ultraviolet light and tobacco smoke [24–28]. Defects in DNA damage repair lead to the accumulation of mutations caused by replicative errors and environmental damage [29, 30]. The core DNA mismatch repair protein complex is composed of two cooperative dimers: the PMS2 protein dimerizes with MLH1 to form the complex MutL-alpha, which cooperates with the MSH2-MSH6 dimer, MutS-alpha, to repair single base pair mismatches and small insertion–deletion loops [31–33]. Perturbations in mismatch repair gene expression, both loss and overexpression, can be deleterious to genomic stability [34–36], and loss of function mutations in mismatch repair pathway genes are known to correlate with high TMB in tumors [37–39]. As such, tumors with defective DNA repair mechanisms are more likely to benefit from immunotherapy [4].

Mutations in DNA damage repair proteins occur as both germline polymorphisms and de novo somatic mutations. Several hereditary cancer syndromes are the result of germline loss of function mutations in mismatch repair pathway genes [40, 41]. In Lynch syndrome, mutations in *MSH2* and *MLH1* are most often observed, with *MSH6* and *PMS2* mutations present in a minority of patients [42]. In all cases, these germline variants lead to the loss of DNA damage repair activity and subsequent hypermutation. Typically, tumorigenesis in these cells occurs after loss of the single functional wild-type copy of the mutated gene. Somatic mutations in DNA mismatch repair genes produce a similar cellular phenotype to tumors with germline defects [43].

DNA replication is another key pathway in which defects can lead to increased somatic mutation rate. Recognition and removal of errors during replication are critical functions of DNA polymerases [44]. POLD1 and POLE are involved in removal of errors during lagging- and leading-strand replication, respectively [44], and mutations in these genes can result in high TMB. The exonuclease domain in both genes is responsible for proofreading activity, and mutations in this domain are associated with hypermutation and tumorigenesis [45, 46]. Somatic loss of function mutations in *POLE* and *POLD1* lead to hypermutation [47, 48]. Loss of *TP53* DNA damage checkpoint activity, by somatic mutation, copy number loss, or epigenetic silencing, increases DNA damage tolerance and can also be associated with increased mutation frequency [49]. Loss of function mutations in *TP53* are very common in cancer and are a somatic marker of elevated mutation rate [50]. Mutations in a number of other genes have also been linked to increased TMB [28, 51], but their function is less well understood. Further understanding the factors associated with increased TMB is important for better understanding this key driver of cancer progression and for understanding the molecular mechanisms which lead to high TMB.

Whole exome sequencing (WES) has been previously used to measure TMB, and TMB levels measured by WES and, in some cases, smaller gene panels have been shown to be associated with response to immunotherapy [52, 53]. The Cancer Genome Atlas (TCGA) project and several other studies have used WES to measure TMB across cancer types and found a wide distribution of TMB across ~20–30 cancer types [28, 51, 54]. Studies focusing on single disease types have shown that high TMB measured from whole exome data is associated with better response rates to immunotherapies in melanoma [21] and non-small cell lung cancer cohorts [20]. Recent studies have also shown that TMB can be accurately measured in smaller gene assays encompassing several hundred genes and that looking at such a panel of genes, the same stratification of patient response based on TMB level exists for some indications [52, 53]. This suggests that a diagnostic assay targeting several hundred genes can accurately measure TMB and that these findings will be clinically actionable.

We sought to better understand the landscape of TMB across the spectrum of human cancer based on data from comprehensive genomic profiling (CGP) of more than 100,000 patient tumors of diverse type. Our analysis expands significantly upon existing data that quantify mutation burden in cancer [28, 51], providing data for many previously undescribed cancer types. We provide new data supporting rational expansion of the patient population that could benefit from immunotherapy and which will allow informed design of clinical trials of immunotherapy agents in untested cancer types. We identify somatically altered genes associated with significantly increased TMB and identify a novel mutation hotspot in the promoter of the *PMS2* gene, which is mutated in ~10% of skin cancers and is associated with greatly increased TMB.

## Methods

### Comprehensive genomic profiling

CGP was performed using the FoundationOne assay (Cambridge, MA, USA), as previously described in detail [55, 56]. Briefly, the pathologic diagnosis of each case was confirmed by review of hematoxylin and eosin stained slides and all samples that advanced to DNA extraction contained a minimum of 20% tumor cells. Hybridization capture of exonic regions from 185, 236, 315, or 405 cancer-related genes and select introns from 19, 28, or 31 genes commonly rearranged in cancer was applied to ≥50 ng of DNA extracted from formalin-fixed, paraffin-embedded clinical cancer specimens. These libraries were sequenced to high, uniform median coverage (>500×) and assessed for base substitutions, short insertions and deletions, copy number alterations, and gene fusions/rearrangements [55]. Data from all versions of the FoundationOne assay were used in the analysis. Hybridization capture baits for *PMS2* are identical across all assay versions.

### WES analysis of TCGA data

WES was performed on 29 samples as previously described [57] for which CGP had also been performed. Briefly, tumors were sequenced using Agilent’s exome enrichment kit (Sure Select V4; with >50% of baits above 25× coverage). The matched blood-derived DNA was also sequenced. Base calls and intensities from the Illumina HiSeq 2500 were processed into FASTQ files using CASAVA. The paired-end FASTQ files were aligned to the genome (to UCSC’s hg19 GRCh37) with BWA (v0.5.9) [58]. Duplicate paired-end sequences were removed using Picard MarkDuplicates (v1.35) to reduce potential PCR bias. Aligned reads were realigned for known insertion/deletion events using SRMA (v0.1.155) [59]. Base quality scores were recalibrated using the Genome Analysis Toolkit (v1.1-28) [60]. Somatic substitutions were identified using MuTect (v1.1.4) [61]. Mutations were then filtered against common single-nucleotide polymorphisms (SNPs) found in dbSNP (v132), the 1000 Genomes Project (Feb 2012), a 69-sample Complete Genomics data set, and the Exome Sequencing Project (v6500).

TCGA data were obtained from public repositories [54]. For this analysis, we used the somatic called variants as determined by TCGA as the raw mutation count. We used 38 Mb as the estimate of the exome size. For the downsampling analysis, we simulated the observed number of mutations/Mb 1000 times using the binomial distribution at whole exome TMB = 100 mutations/Mb, 20 mutations/Mb, and 10 mutations/Mb and did this for megabases of exome sequenced ranging from 0–10 Mb. Melanoma TCGA data were obtained from dbGap accession number phs000452.v1.p1 [62].

### Cohort selection

From an initial clinical cohort of 102,292 samples, duplicate assay results from the same patient were excluded, and samples with less that 300× median exon coverage were excluded to make an analysis set of 92,439 samples. For analyses by cancer type, they must contain a minimum of 50 unique specimens following sample level filtering.

### Tumor mutational burden

TMB was defined as the number of somatic, coding, base substitution, and indel mutations per megabase of genome examined. All base substitutions and indels in the coding region of targeted genes, including synonymous alterations, are initially counted before filtering as described below. Synonymous mutations are counted in order to reduce sampling noise. While synonymous mutations are not likely to be directly involved in creating immunogenicity, their presence is a signal of mutational processes that will also have resulted in nonsynonymous mutations and neoantigens elsewhere in the genome. Non-coding alterations were not counted. Alterations listed as known somatic alterations in COSMIC and truncations in tumor suppressor genes were not counted, since our assay genes are biased toward genes with functional mutations in cancer [63]. Alterations predicted to be germline by the somatic-germline-zygosity algorithm were not counted [64]. Alterations that were recurrently predicted to be germline in our cohort of clinical specimens were not counted. Known germline alterations in dbSNP were not counted. Germline alterations occurring with two or more counts in the ExAC database were not counted [65]. To calculate the TMB per megabase, the total number of mutations counted is divided by the size of the coding region of the targeted territory. The nonparametric Mann–Whitney U-test was subsequently used to test for significance in difference of means between two populations.

### Microsatellite instability

Microsatellite instability calling was performed on 62,150 samples, and analyses comparing MSI to TMB were limited to samples where both MSI status and TMB were determined.

To determine MSI status, 114 intronic homopolymer repeat loci with adequate coverage on the CGP panel were analyzed for length variability and compiled into an overall MSI score via principal components analysis.

The 114 loci were selected from a total set of 1897 that have adequate coverage on the FMI FoundationOne bait set. Amongst the 1897 microsatellites, the 114 that maximized variability between samples were chosen. Each chosen locus was intronic and had hg19 reference repeat length of 10–20 bp. This range of repeat lengths was chosen such that the microsatellites are long enough to produce a high rate of DNA polymerase slippage, while short enough such that they are well within the 49-bp read length of next-generation sequencing to facilitate alignment to the human reference genome. Translation of the MSI score to MSI-H or MSS (MSI-Stable) was established using a training data set.

Using the 114 loci, for each training sample the repeat length in each read that spans the locus was calculated. The means and variances of repeat lengths across the reads were recorded, forming 228 data points per sample. We then used principal components analysis to project the 228-dimension data onto a single dimension (the first principal component) that maximized the data separation, producing a next-generation sequencing-based “MSI score”. There was no need to extend beyond the first principal component, as it explained ~50% of the total data variance, while none of the other principal components explained more than 4% each. Ranges of the MSI score were assigned MSI-High (MSI-H), MSI-ambiguous, or microsatellite stable (MSS) by manual unsupervised clustering of specimens for which MSI status was previously assessed either via IHC if available or approximated by the number of homopolymer indel mutations detected by our standard pipeline.

### Statistical association testing

To test for statistical association between genes and tumor mutation burden, we counted known and likely functional short variants in each gene, excluding mutations that occurred in homopolymers of length 6 or greater. We tested for association for all genes with six or more specimens with mutations that passed our filtering. We added a pseudo-count to each TMB value. We then fit a linear model of the type log_10_(TMB) ~ functional mutation status + disease type. We used the factor loading coefficient to determine the genes with the greatest effect size. This coefficient gives the change in log_10_(TMB) between samples with presence or absence of a functional mutation in that gene, while holding the disease type constant. We chose an effect size (factor loading) cutoff of 0.5, which when converted back from log space is equivalent to a 3.1-fold increase in TMB compared to wild-type TMB (3.6 mutations/Mb).

To test for association between alterations and tumor mutation burden, we tested all short variants occurring at a frequency of greater than 1 per 2000 specimens, excluding mutations that occurred in homopolymers of length 6 or greater and filtering out mutations present in dbSNP. We then fit a linear model, as above, of the type log_10_(TMB) ~ alteration status + disease type. For both tests, we corrected for multiple testing using the false discovery rate (FDR) method [66].

### Co-occurrence

We tested for co-occurrence of functional gene mutations with *PMS2* promoter mutations using logistic regression. We fit a model of the type: status of *PMS2* promoter mutations in melanoma ~ gene functional mutation status + TMB. We then corrected for multiple testing using the FDR method [66].

## Results

### TMB can be accurately measured by a targeted comprehensive genomic profiling assay

**Fig. 1:**
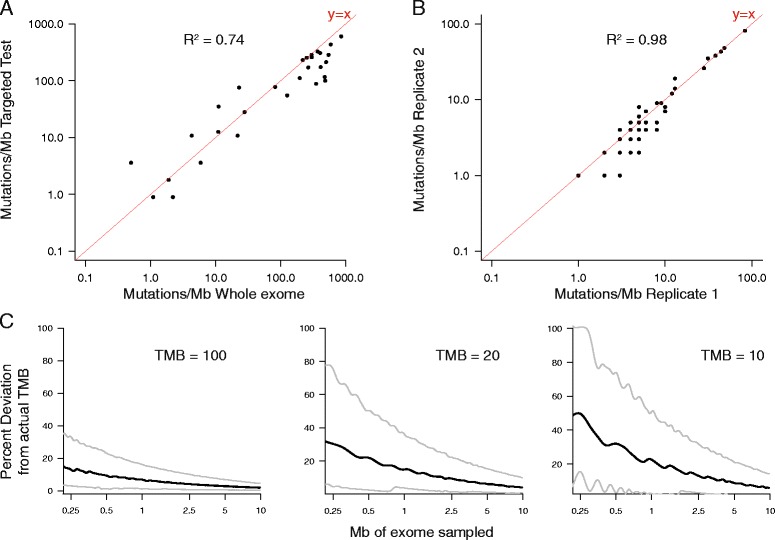
Accuracy and precision of comprehensive genomic profiling for assessing tumor mutation burden. **a** Comparison of tumor mutation burden measured by whole exome sequencing versus comprehensive genomic profiling. Tumor mutation burden (mutations/Mb) was measured in 29 samples by whole exome sequencing of matched tumor and normal samples and by comprehensive genomic profiling (see “Methods” for more details). The line y = x is plotted in *red*. **b** Tumor mutation burden measured by comprehensive genomic profiling in 60 pairs of replicates. The line y = x is plotted in *red*. **c** Results of simulations of percentage deviation from actual TMB when sampling different numbers of megabases sequenced. Median observed deviation is shown in *black* and 10% and 90% confidence interval are shown in *grey*. Lines are smoothed using a cubic smoothing spline with smoothing parameter = 0.6. *Left*: results of simulations with TMB equal to 100 mutations/Mb. *Center*: results of simulations with TMB equal to 20 mutations/Mb. The median line was smoothed with smoothing parameter = 0.8. *Right*: results of simulations with TMB equal to 10 mutations/Mb. The median line was smoothed with smoothing parameter = 0.8

We first sought to determine whether TMB, as measured by a comprehensive genomic profiling (CGP) assay targeting 315 genes (1.1 Mb of coding genome), could provide an accurate assessment of whole exome TMB. We performed targeted CGP and WES on the same biopsy specimen for a cohort of 29 tumors. From both the WES and targeted CGP samples, we calculated the number of somatic, coding, base substitution, and indel mutations per megabase of interrogated genome (see “Methods”). For the WES samples, tumor and normal tissue were each sequenced in order to distinguish germline polymorphisms from somatic mutations. For the targeted CGP samples, no matched normal material was sequenced; rather, genomic variants were stringently filtered to eliminate germline polymorphisms (see “Methods” for details). We found that the tumor mutation burden calculated by these two methods was highly correlated (R^2^ = 0.74; Fig. 1a).

We also assessed the reproducibility of our method for calculating TMB using targeted CGP. For 60 samples for which CGP was performed more than once, we compared the TMB between replicates. We found that these values were highly correlated (R^2^ = 0.98), indicating that this method for measuring TMB has high precision (Fig. 1b).

We finally sought to determine the effects of sequencing different amounts of the genome and how that might affect our ability to accurately determine TMB. We sampled the number of mutations that we would expect to see at different TMB levels (100 mutations/Mb, 20 mutations/Mb, 10 mutations/Mb) and at different amounts of megabases sequenced, from 0.2 to 10 Mb, 1000 times for each TMB level and sequencing amount. For each sample, we then measured the percentage deviation from the whole exome TMB (Fig. 1c). We found that, as expected, the percentage deviation is lower for high underlying TMB, meaning that specimens with high TMB can be effectively identified by targeted sequencing of several hundred genes. In contrast, for intermediate levels of TMB, the percentage deviation starts to increase, especially with less than 0.5 Mb sequenced (Fig. 1c).

We also analyzed whole-exome sequencing data from 35 studies, published as part of TCGA, examining a total of 8917 cancer specimens [54]. We determined the number of mutations in total and compared that to the number of mutations in the 315 genes targeted by our assay. As expected, these results were also highly correlated (R^2^ = 0.98). These results demonstrate that CGP targeting the entire coding region of several hundred genes can accurately assess whole exome mutational burden.

### The landscape of mutation burden across cancer types

We next examined the landscape of TMB across the cohort of patients profiled in our laboratory. CGP was performed in the course of routine clinical care for 102,292 samples (see “Methods”). The unique patient cohort contained 41,964 male and 50,376 female patients. Median patient age at the time of specimen collection was 60 years (range <1 year to >89 years), and 2.5% of cases were from pediatric patients under 18 years old. This body of data provided 541 distinct cancer types for analysis. Notably, the majority of specimens were from patients with significantly pre-treated, advanced, and metastatic disease. Across the entire dataset, the median TMB was 3.6 mutations/Mb, with a range of 0–1241 mutations/Mb. This agrees well with previous estimates of mutation burden from whole exome studies [28, 51]. We found a significant increase in TMB associated with increased age (*p* < 1 × 10^–16^), though the effect size was small (Additional file 1: Figure S1). Median TMB at age 10 was 1.67 mutations/Mb, and median TMB at age 88 was 4.50 mutations/Mb. A linear model fit to the data predicted a 2.4-fold difference in TMB between age 10 and age 90, consistent with the median TMB differences at these ages. There was no statistically significant difference in median TMB between female and male patients (Additional file 2: Figure S2).

**Fig. 2:**
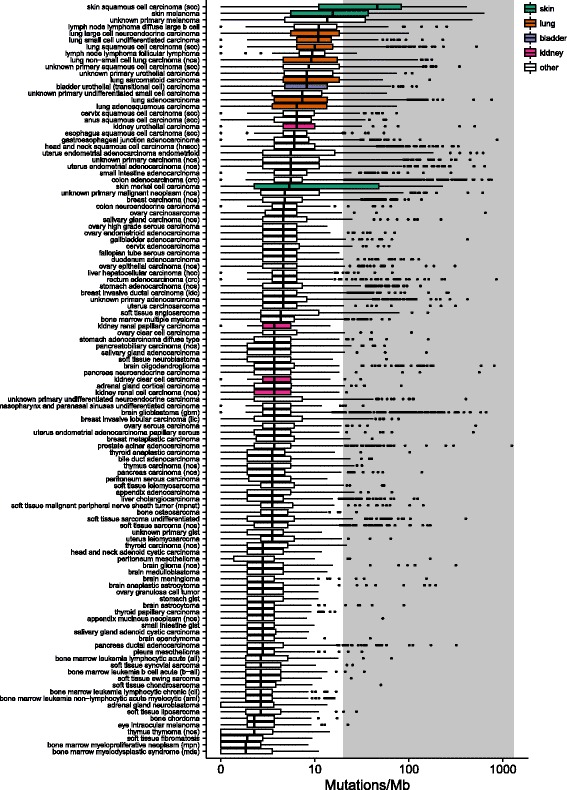
The landscape of tumor mutation burden. For all disease types with greater than 100 samples, the median mutation burden is plotted for each disease type. The *left* and *right* edges of the boxes correspond to the 25th and 75th percentiles. Whiskers extend to the highest value that is within 1.5 × IQR of the hinge, where IQR is the inter-quartile range, or distance between the first and third quartiles. Points beyond this are plotted individually. Tissue types of interest are shown in color, as follows: skin, *green*; lung, *orange*; bladder, *purple*; kidney, *pink*; other, *white*. The area above 20 mutations/Mb, which we have designated as high TMB, is colored in *grey*

We examined TMB for 167 distinct cancer types for which we had tested more than 50 specimens (Fig. 2; Additional file 3: Table S1). The median TMB ranged widely, from 0.8 mutations/Mb in bone marrow myelodysplastic syndrome to 45.2 mutations/Mb in skin squamous cell carcinoma. As expected, we found that pediatric malignancies (patient age less than 18 years) had lower TMB (median 1.7 mutations/Mb) than adult malignancies (median 3.6 mutations/Mb). Disease types common in pediatric patients, such as leukemia, lymphoma, and neuroblastoma, had low TMB, as did sarcomas (Additional file 3: Table S1). The relationship between TMB and age also differed across disease types (Additional file 4: Figure S3).

Diseases known to have significant mutagen exposure, such as lung cancers and melanoma, were more highly mutated (median TMB 7.2 mutations/Mb and 13.5 mutations/Mb, respectively). Disease indications in which immunotherapies are currently approved, including melanoma, non-small cell lung cancer (NSCLC), and bladder, had high TMB, as expected (Additional file 3: Table S1). Identifying additional cancer types with high TMB may represent an opportunity to expand the list of indications that respond favorably to immune checkpoint blockade. These include skin squamous cell carcinoma, lung small cell undifferentiated carcinoma, diffuse large B cell lymphoma, as well as many other types of cancer (Fig. 1). In addition to identifying additional cancer types with high overall TMB, we also found cases with high TMB across nearly every cancer type (Table 1; Additional file 3: Table S1). This raises the possibility that patients with high TMB who may benefit from immunotherapy can be identified in nearly every type of cancer. For example, in soft tissue angiosarcoma, while the median mutation burden was 3.8 mutations/Mb, 13.4% of cases had more than 20 mutations/Mb. Overall, we identified 20 tumor types affecting eight tissues with greater than 10% of patients who had high TMB and 38 tumor types affecting 19 tissues with greater than 5% of patients with high TMB (Table 1).

**Table 1:** Disease indications with greater than 5% of specimens showing high TMB (>20 mutations/Mb)

Disease type	Specimen count	Median mutations/Mb	Percentage cases with >20 mutations/Mb (95% CI)
Skin basal cell carcinoma	92	47.3	70.7 (60.7–79)
Skin squamous cell carcinoma (SCC)	266	45.2	67.3 (61.4–72.7)
Skin melanoma	879	14.4	39.7 (36.4–42.9)
Skin merkel cell carcinoma	206	4.3	37.9 (31.5–44.7)
Unknown primary melanoma	1324	12.6	37.6 (35–40.2)
Head and neck melanoma	59	6.3	25.4 (14.7–36)
Lung large cell carcinoma	74	12.2	24.3 (14.9–33.7)
Unknown primary squamous cell carcinoma (SCC)	606	7.6	21.6 (18.4–24.9)
Lung large cell neuroendocrine carcinoma	288	9.9	19.8 (15.6–24.8)
Lung sarcomatoid carcinoma	130	7.2	19.2 (12.7–26)
Stomach adenocarcinoma intestinal type	58	5.0	19 (10.9–30.9)
Uterus endometrial adenocarcinoma endometrioid	459	4.5	18.5 (15–22.1)
Lymph node lymphoma diffuse large B cell	348	10.0	18.4 (14.7–22.8)
Lung non-small cell lung carcinoma (NOS)	2636	8.1	17 (15.6–18.5)
Unknown primary sarcomatoid carcinoma	64	5.4	15.6 (7.6–24.6)
Unknown primary malignant neoplasm (NOS)	491	3.8	14.9 (12–18.3)
Uterus endometrial adenocarcinoma (NOS)	743	4.5	14.7 (12.3–17.4)
Bladder carcinoma (NOS)	77	8.1	14.3 (8.2–23.8)
Unknown primary urothelial carcinoma	188	7.2	13.8 (9.2–18.9)
Soft tissue angiosarcoma	157	3.3	13.4 (8.9–19.6)
Lung adenocarcinoma	11855	6.3	12.3 (11.7–12.9)
Lung adenosquamous carcinoma	154	5.4	12.3 (7.5–17.7)
Skin adnexal carcinoma	74	3.6	12.2 (6.5–21.5)
Bladder urothelial (transitional cell) carcinoma	1218	7.2	11.9 (10.1–13.8)
Lymph node lymphoma B-cell (NOS)	88	6.3	11.4 (6.3–19.7)
Lung squamous cell carcinoma (SCC)	2102	9.0	11.3 (10–12.7)
Unknown primary carcinoma (NOS)	1405	4.5	10.7 (9.2–12.4)
Head and neck squamous cell carcinoma (HNSCC)	1184	5.0	10.1 (8.5–11.9)
Lung small cell undifferentiated carcinoma	913	9.9	9 (7.3–11)
Nasopharynx and paranasal sinuses squamous cell Carcinoma (SCC)	67	4.5	9 (4.2–18.2)
Ovary endometrioid adenocarcinoma	105	3.6	8.6 (4.6–15.5)
Unknown primary undifferentiated small cell carcinoma	117	6.3	8.5 (4.1–14)
Brain oligodendroglioma	321	2.7	8.4 (5.6–11.6)
Small intestine adenocarcinoma	277	4.5	8.3 (5.3–11.7)
Soft tissue malignant peripheral nerve sheath tumor (MPNST)	134	2.5	8.2 (4.1–13.2)
Soft tissue sarcoma undifferentiated	260	2.5	8.1 (5.3–12)
Uterus endometrial adenocarcinoma clear cell	62	3.6	8.1 (3.5–17.5)
Prostate undifferentiated carcinoma	91	3.6	7.7 (3.8–15)
Salivary gland mucoepidermoid carcinoma	55	2.7	7.3 (2.9–17.3)
Unknown primary adenocarcinoma	2751	3.6	6.9 (6–7.9)
Ureter urothelial carcinoma	88	5.4	6.8 (2.5–12.6)
Cervix squamous cell carcinoma (SCC)	284	5.4	6.7 (4.3–10.2)
Penis squamous cell carcinoma (SCC)	60	4.5	6.7 (2.6–15.9)
salivary gland carcinoma (NOS)	160	3.6	6.3 (3.4–11.1)
Kidney urothelial carcinoma	224	5.4	6.3 (3.8–10.2)
Unknown primary undifferentiated neuroendocrine carcinoma	674	2.7	6.1 (4.5–8.1)
Duodenum adenocarcinoma	249	3.6	6 (3.4–9.2)

*CI* confidence interval, *NOS* not otherwise specified

### TMB and microsatellite instability

**Fig. 3:**
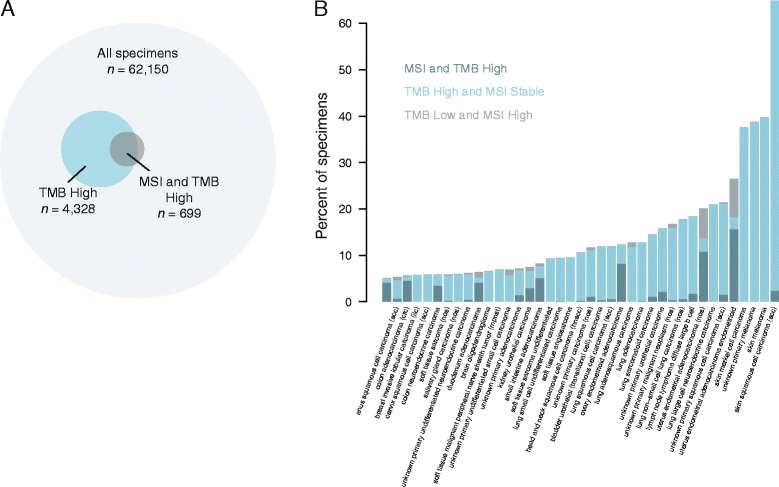
The relationship between tumor mutation burden and microsatellite instability. **a** Specimens for which we measured both TMB and microsatellite instability. MSI calls were only available for 62,150 samples from the most recent versions of the assay. Specimens with TMB low and called as MSI-Stable are shown in *light grey*, specimens with high TMB (mutations/Mb >20) are shown in *blue*, and specimens called as MSI-High are shown in *dark grey*. **b** The proportion of samples called as MSI and TMB high (*dark blue*), TMB high and MSI-Stable (*light blue*), and TMB low and MSI-High (*grey*) for each of the disease types with greater than 0.3% of samples called as either TMB or MSI-High

Microsatellite instability is another marker of genomic instability. We characterized microsatellite instability in a subset of our cohort and classified samples as MSI-High (microsatellite instability high) or MS-Stable (microsatellite stable) (see “Methods”; n = 62,150). We found that microsatellite instability (MSI-High) generally occurred as a subset of high TMB (Fig. 3a). The vast majority of MSI-High samples also had high TMB (83%), and 97% had TMB ≥10 mutations/Mb. However, the converse was not true; only 16% of samples with high TMB were classified as MSI-High. The co-occurrence of these two phenotypes was highly dependent on the cancer type. In gastrointestinal cancers such as stomach adenocarcinoma, duodenum adenocarcinoma, and small intestine adenocarcinoma, MSI-High and high TMB almost always co-occur, while in melanoma, squamous cell carcinoma, and lung carcinoma, high TMB was fairly common but MSI-High was very uncommon (Fig. 3b).

### Identifying known genes and alterations associated with increased TMB

**Fig. 4:**
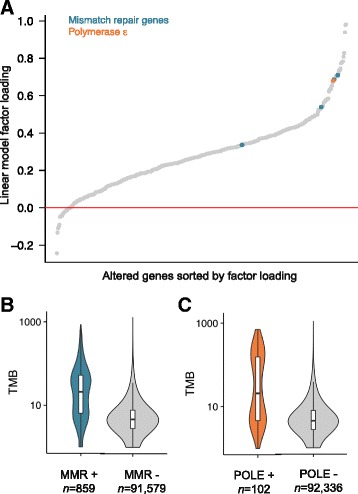
Associating mutations in cancer genes with tumor mutational burden. **a** Coefficient from linear model. Genes are sorted by this ratio. Genes involved in mismatch repair (*MSH2*, *MSH6*, *MLH1*, *PMS2*) are highlighted in *blue*. DNA polymerase ε (*POLE*) is highlighted in *orange*. **b** Plot of mutation burden in specimens with known or likely driver mutations in any of the mismatch repair genes listed above (*MMR+*), *n* = 859, and of specimens without such a mutation (*MMR*−), *n* = 91,579. **c** Plot of mutation burden in specimens with known or likely driver mutations in POLE (*n* = 102) and specimens without such mutations (*n* = 92,336)

In order to investigate the molecular basis of high TMB across our samples, we performed statistical analysis to identify the genes or specific mutations whose presence was associated with increased TMB. We first tested whether the presence of any functional alterations (base substitutions or short indels) in each of the targeted genes was associated with TMB (see “Methods”), controlling for cancer type. We found 257 genes which were significantly associated with TMB at FDR = 0.0001. This is not entirely surprising, as specimens with high TMB would be expected to have a greater number of functional oncogenic mutations. Many of these genes were associated with relatively small increases in TMB after controlling for disease type (Fig. 4a). Consequently, we focused on the statistically significant effects with the greatest magnitude. We identified 48 genes significantly associated and with factor loading >0.5 (see “Methods”; Fig. 4a; Additional file 5: Table S2).

Genes associated with large increases in TMB include known DNA mismatch repair pathway genes (*MSH2*, *MSH6*, *MLH1*, *PMS2*) and DNA polymerases (*POLE*) (Fig. 4a–c). (Additional file 5: Table S2). Across the cohort, functional mutations in these mismatch repair genes and DNA polymerase occur in 13.5% of the cases with high TMB (858 cases with known functional mutations in mismatch repair or *POLE* out of the 6348 cases with high tumor mutation burden). Many of the mutations found were inactivating frameshift alterations, and *MSH6* was the most frequently mutated (Additional file 6: Figure S4). We found mismatch repair mutations to be particularly common in skin squamous cell carcinoma (6.7%), uterus endometrial adenocarcinoma, subtype not otherwise specified; (6.0% of cases), and uterus endometrial adenocarcinoma endometrioid (5.8%). Our results are consistent with the known role of alterations in mismatch repair genes in leading to hypermutation.

In order to identify potential novel mutations associated with increased mutation rate, we also tested for association between TMB and all genomic alterations in our dataset (see “Methods”). We identified 117 somatic mutations significantly associated with increased tumor mutation burden at FDR = 0.05 and with factor loading >0.15 (Additional file 7: Table S3). As expected, many statistically significant mutations occurred in mismatch repair genes, and *POLE* P286R, a genomic alteration that is known to cause hyper-mutant cancers [67], was the second most significant (*p* = 1.1 × 10^–72^).

### Novel promoter mutations in *PMS2* are associated with high mutation burden and occur frequently in melanoma

**Fig. 5:**
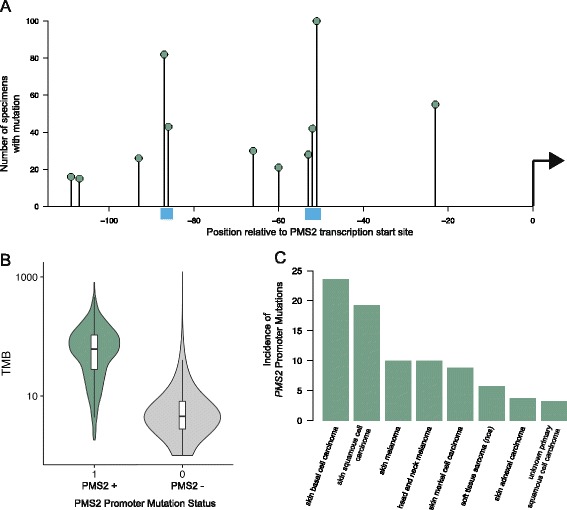
Recurrent *PMS2* mutations are associated with increased mutation burden and are stratified by disease type. **a** Location of recurrent *PMS2* promoter mutations upstream of the transcription start site. Locations showing multiple dinucleotide events are marked with a *blue box*. **b** Mutation burden in *PMS2* mutant versus wild-type specimens. For the indicated disease and selected mutation or collection of mutations, tumors were classified as Mut + or Mut−. Mutation burden for these two sample populations is plotted. Whiskers extend to the highest value that is within 1.5 × IQR of the hinge, where IQR is the inter-quartile range, or distance between the first and third quartiles. Points beyond this are not shown. **c** Percentage of specimens with *PMS2* promoter mutations in select disease types. The percentage of specimens with any of the *PMS2* promoter mutations is plotted

In addition to previously known mutations, we identified a cluster of somatic mutations in the promoter region, ~50–100 bp upstream of the transcription start site of the *PMS2* gene that were significantly associated with a large increase in TMB. The most statistically significant mutation was a chr7:6048788:C > T (*p* = 1.2 × 10^–49^). Melanoma specimens harboring this mutation showed a 5.3× increase in median TMB compared to specimens that did not harbor this mutation. In total, we identified 12 positions within the promoter of the *PMS2* gene which were recurrently mutated and associated with increased mutation burden (Fig. 5a; Additional file 8: Table S4). The original mutation identified was frequently mutated as part of a dinucleotide substitution (chr7:6048788-6048789:CC > TT). The presence of any one of the *PMS2* promoter alterations was associated with a 5.3-fold increase in median TMB when compared with PMS2 promoter wild-type samples in melanoma specimens (Fig. 5b). This increase in the median TMB of samples harboring promoter mutations is comparable in magnitude to the increase in mutation burden in specimens with functional mutations in the coding region of DNA repair pathway genes *MSH2*, *MSH6*, *MLH1*, and *PMS2* (Fig. 3). Mutations in the coding regions of *PMS2* were less frequent (0.2%, 191/92438) than mutations in the promoter region.

These *PMS2* promoter mutations occurred frequently in melanoma, in 10.0% of cases (173/1731). They were also found frequently in skin basal cell carcinoma (23%, 17/72 specimens) and skin squamous cell carcinoma (19%, 39/203 specimens) and less frequently in several other tumor types (Additional file 9: Table S5). We tested for co-occurrence of *PMS2* promoter mutations with mutations in other genes in melanoma. After controlling for TMB (see “Methods”), we found that no other mutations significantly co-occurred (Additional file 10: Table S6).

To confirm that *PMS2* promoter mutations were somatic in origin, we carried out several analyses. We first looked in TCGA whole exome data from 50 melanoma patients and confirmed the somatic status of three of the mutations found in our cohort (chr7:6048723, chr7:6048760, and chr7:6048824) [62]. In this dataset, the frequency of the three PMS2 promoter mutations listed above is similar to the frequency of all PMS2 promoter mutations found in our data and significantly associated with TMB (4/50, 8.0%, 95% confidence interval (CI) 3.1–18.8%, and 10.0%, 95% CI 8.6–11.5%, respectively). We also queried public germline databases dbSNP142 and ExAC, and none of the *PMS2* promoter mutations associated with high mutation burden were found in either database. Finally, we used an algorithm that uses the mutation allele frequency and genome-wide copy number model of genomic alterations to determine their germline or somatic origin (see “Methods”). We found that of the variants which were able to be called as somatic or germline, 274 of the variants out of 294 (93.1%) were called as somatic (Additional file 11: Table S7). Furthermore, the median allele frequency of *PMS2* promoter mutations in melanoma is 0.26 (range 0.05–0.85), which is lower than that for *BRAF* V600 mutations occurring in the same tumor type (median 0.37, max 0.97; Additional file 12: Table S8). These data demonstrate that these *PMS2* promoter mutations are most frequently somatic in origin. Finally, we used several computational methods to assess the functional impact of these mutations [68–70], using methods which integrate conservation information as well as multiple functional genomics data from ENCODE such as DNase I patterns and transcription factor binding (Additional file 13: Table S9). Interestingly, these methods agree in terms of which of the mutations we identified are most likely to be functional; chr7:6048760 and chr7:6048824 consistently had the most significant functional scores.

## Discussion

We have shown that tumor mutation burden calculated using a 1.1-Mb CGP assay agrees well with whole exome measures of mutation burden. This indicates that CGP, targeting the entire coding region of several hundred genes, covers sufficient genomic space to accurately assess whole exome mutational burden. We found that filtering out germline alterations and rare variants was important to obtaining accurate measurements of TMB, and this will especially be important in patients from ethnic backgrounds not well represented in sequencing datasets. These findings indicate that CGP is an accurate, cost-effective, and clinically available tool for measuring TMB. The results of our downsampling analysis show that the variation in measurement due to sampling when sequencing 1.1 Mb is acceptably low, resulting in highly accurate calling of TMB at a range of TMB levels. This sampling variation increases as the number of megabases sequenced decreases, especially at lower levels of TMB. While targeted CGP can be used to accurately assess TMB, it is not currently suited for identification of neoantigens, which might occur in any gene.

We characterized and provide extensive data describing tumor mutational burden across more than 100,000 clinical cancer specimens from advanced disease, including many previously undescribed types of cancer. These data should help to guide design of immunotherapy clinical trials across a broader range of indications. Currently, immunotherapies targeting CTLA-4, PD-1, and PD-L1 are approved in a small number of indications, melanoma, bladder, NSCLC, and renal cell carcinoma. Not surprisingly, we observe that melanoma and NSCLC represent some of the highest mutation burden indications. We identified several novel disease types with high TMB which may be good targets for immuno-oncology treatment development. In addition, we observed a wide range of TMB across many cancer types, similar to findings from previous studies [28, 51]. We have found that there may be many disease types with a substantial portion of patients who might benefit from these therapies. Overall, we identified 20 tumor types affecting eight tissues where greater than 10% of patients had high TMB.

Understanding the factors associated with genomic instability is also important to better understand carcinogenesis and progression. We characterized the distribution and prevalence of coding mutations in known genes involved in mismatch repair and DNA replication. However, overall mutations in these genes accounted for less than 10% of cases with high TMB. We also identified several other genes associated with high TMB. Alterations in *TOP2A* were associated with a large increase in TMB, although we only identified eight cases of single nucleotide substitutions in this gene. *TP53BP1*, another of the genes showing large effect size, is involved in double-stranded break repair and also implicated in resistance mechanisms [71, 72].

Non-coding mutations have increasingly been found to have a functional role in cancer [73–75]. Our analysis of mutations that are significantly associated with increased tumor mutation burden resulted in the discovery of novel recurrent mutations in the promoter region of mismatch repair pathway gene *PMS2*. We have not definitively shown that these mutations are causal, and additional experiments will be needed to elucidate the function of these promoter mutations. *PMS2* promoter mutations are present in ~10% of melanoma samples and ~8% of squamous cell carcinomas, meaning that, if functional, these mutations may comprise a meaningful subset of alterations in both of these diseases.

## Conclusions

These results show that CGP targeting ~1.1 Mb of coding genome can accurately assess TMB compared with sequencing the whole exome. Using this method, we find that many disease types have a substantial portion of patients with high TMB who might benefit from immunotherapy. Finally, we identify novel, recurrent promoter mutations in *PMS2* which may be another example of regulatory mutations contributing to tumorigenesis.

## Additional files

Additional file 1: Figure S1. TMB increases with age in adult patients (pdf). TMB values are plotted versus age. The *red line* shows the fit from a linear regression model. (PDF 1455 kb)

Additional file 2: Figure S2. TMB by gender (pdf). TMB for female (*left*) and male (*right*). The *bottom* and *top edges* of the boxes correspond to the 25th and 75th percentiles. Whiskers extend to the highest value that is within 1.5 × IQR of the hinge, where IQR is the inter-quartile range, or distance between the first and third quartiles. Points beyond this are plotted individually. (PDF 23 kb)

Additional file 3: Table S1. Summary of TMB properties by disease (xls). Specimen count, median TMB, maximum TMB, percentage of cases with TMB >20 mutations/Mb, and 95% binomial confidence intervals on the percentage of cases with TMB >20 are provided. (XLSX 18 kb)

Additional file 4: Figure S3. TMB by age in select disease types (pdf). TMB versus age is plotted for select disease types, lung adenocarcinoma, skin squamous cell carcinoma, and colon adenocarcinoma. The *red line* shows the fit from a linear regression model for that disease type. (PDF 940 kb)

Additional file 5: Table S2. TMB association results by gene (xls). *P* value, factor loading coefficient, and the number of specimens with a known or likely functional mutation in the gene are provided. (XLSX 40 kb)

Additional file 6: Figure S4. Location of known or likely functional mutations in mismatch repair and POLE genes (pdf). For the genes *MSH6*, *MLH1*, *MSH2*, *PMS2*, and *POLE*, the count of mutations at each position in the transcript is plotted. (PDF 37 kb)

Additional file 7: Table S3. TMB association results by mutation (xls). The effect of the mutation on the transcript, *p* value, factor loading coefficient, and number of times the mutation was found are provided for each mutation tested for association with TMB for which the corrected *p* value was <0.05. (XLSX 81 kb)

Additional file 8: Table S4. Summary of PMS2 promoter mutations (xls). For each PMS2 promoter mutation which was found to be significantly associated with TMB, the genomic coordinate, number of observations, and median TMB in specimens with that mutation is provided. (XLSX 42 kb)

Additional file 9: Table S5. Disease distribution of PMS2 promoter mutations (xls). The frequency of PMS2 promoter mutations in disease types with frequency greater than 0.1. Frequency, binomial 95% confidence interval on the frequency, number of specimens in that disease type, and number and specimens with promoter mutations is provided. (XLSX 31 kb)

Additional file 10: Table S6. Co-occurrence of PMS2 promoter mutations with alterations in genes (xls). Results of logistic regression test for co-occurrence of alterations with PMS2 promoter mutations in skin melanoma. The mutation count (number of specimens with known or likely functional mutation in the gene), gene mutation frequency in PMS2+ (frequency of known or likely functional mutations in the gene in specimens with PMS2 promoter mutation), gene mutation frequency in PMS2− (frequency of known or likely functional mutations in the gene in specimens without PMS2 promoter mutation), odds ratio, and *p* value are provided. (XLSX 48 kb)

Additional file 11: Table S7. Somatic/germline calls for PMS2 promoter mutations (xls). Results of algorithm to call mutations as somatic, germline, or ambiguous (see “Methods”). For each PMS2 promoter mutation, the number of times it was called somatic, ambiguous, or germline is provided. (XLSX 34 kb)

Additional file 12: Table S8. Allele fraction of PMS2 promoter mutations and selected mutations in melanoma (xls). For selected mutation in melanoma (NRAS Q61K and BRAF V600E), and for each of the PMS2 promoter mutations, the median, minimum, and maximum allele fraction (fraction of reads at that position showing the mutation) and number of specimens with that mutation are provided. (XLSX 40 kb)

Additional file 13: Table S9. Functional scores for PMS2 promoter mutations. For each of the PMS2 promoter mutation locations, the scores for three functional prediction methods are provided. See “References” section. (XLSX 36 kb)

## Abbreviations

CGP: Comprehensive genomic profiling
FDR: False discovery rate
NSCLC: Non-small cell lung cancer
TCGA: The cancer genome atlas
TMB: Tumor mutational burden
WES: Whole exome sequencing
