# Supplementary figures and images for: Analysis of 100,000 human cancer genomes reveals the landscape of tumor mutational burden
Source: Genome Med. 2017 Apr 19;9:34. doi: 10.1186/s13073-017-0424-2 (PMC5395719; doi:10.1186/s13073-017-0424-2)

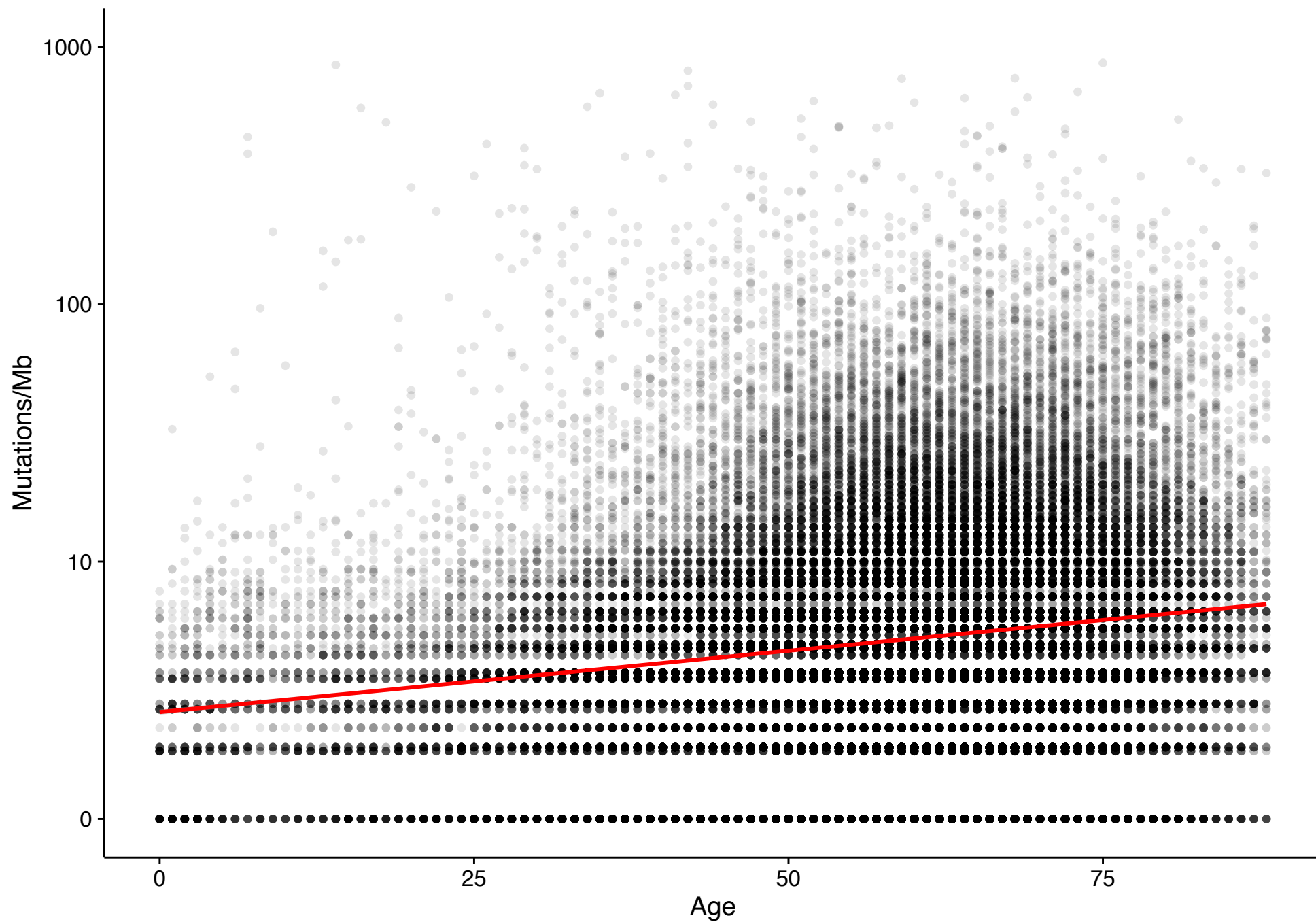

Supplement: Supplementary file 1 — TMB increases with age in adult patients (pdf). TMB values are plotted versus age. The red line shows the fit from a linear regression model. (PDF 1455 kb) [file 13073_2017_424_MOESM1_ESM.pdf]

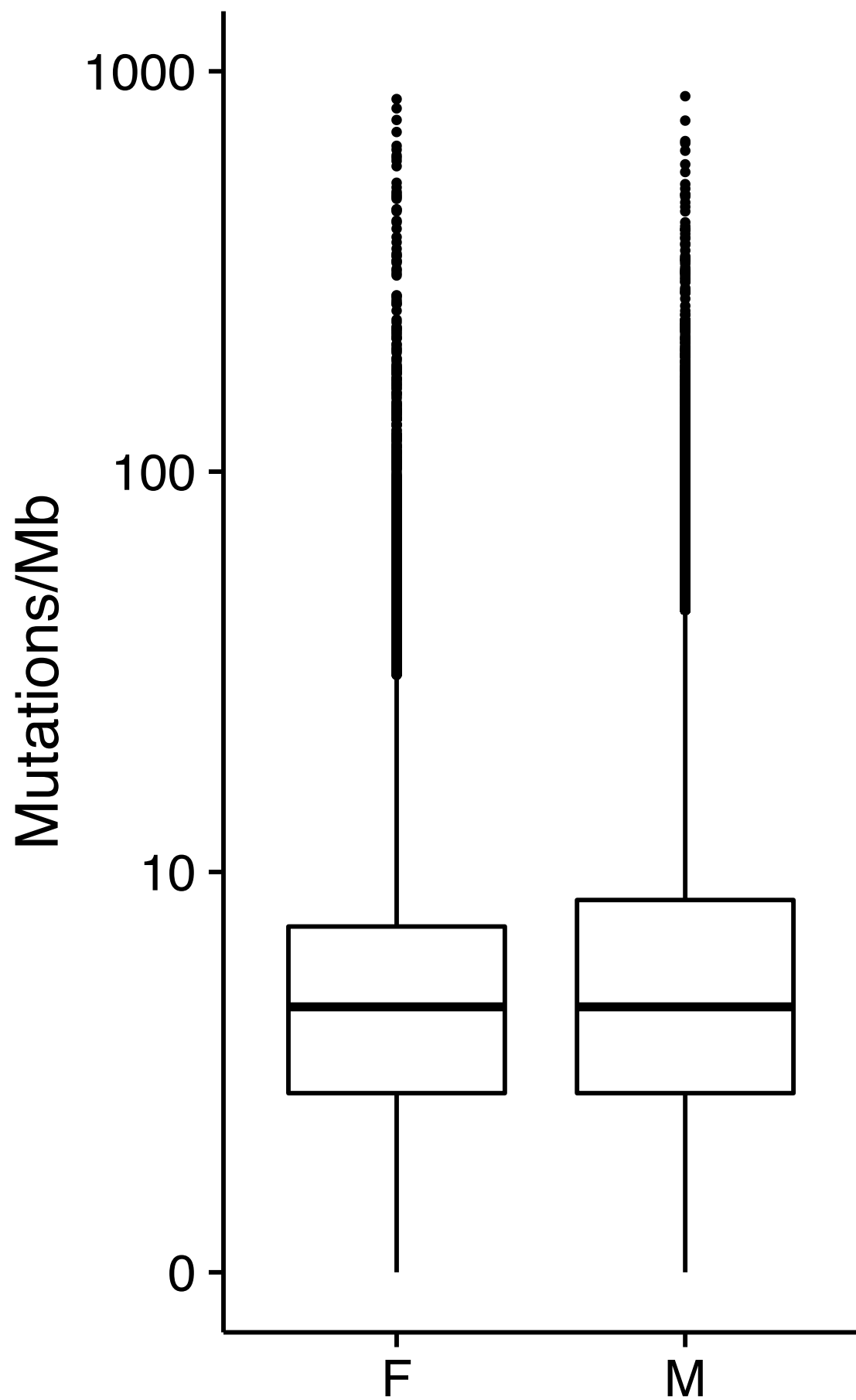

Supplement: Supplementary file 2 — TMB by gender (pdf). TMB for female (left) and male (right). The bottom and top edges of the boxes correspond to the 25th and 75th percentiles. Whiskers extend to the highest value that is within 1.5 × IQR of the hinge, where IQR is the inter-quartile range, or distance between the first and third quartiles. Points beyond this are plotted individually. (PDF 23 kb) [file 13073_2017_424_MOESM2_ESM.pdf]

Lung adenocarcinoma

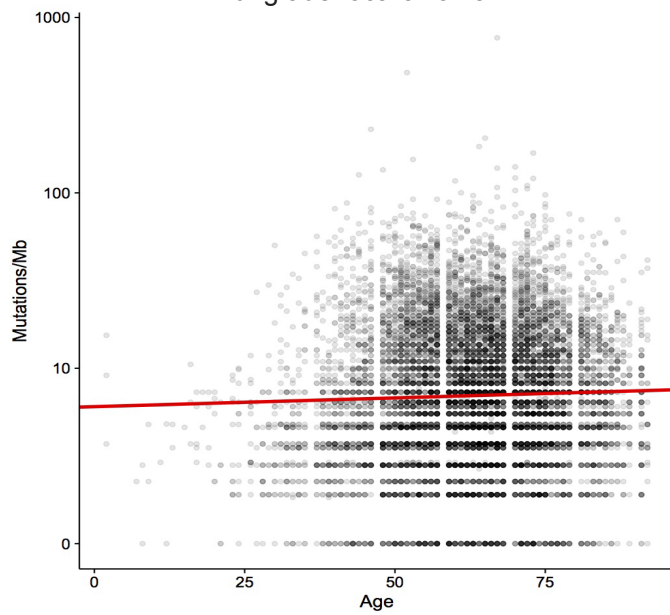

Skin squamous cell carcinoma

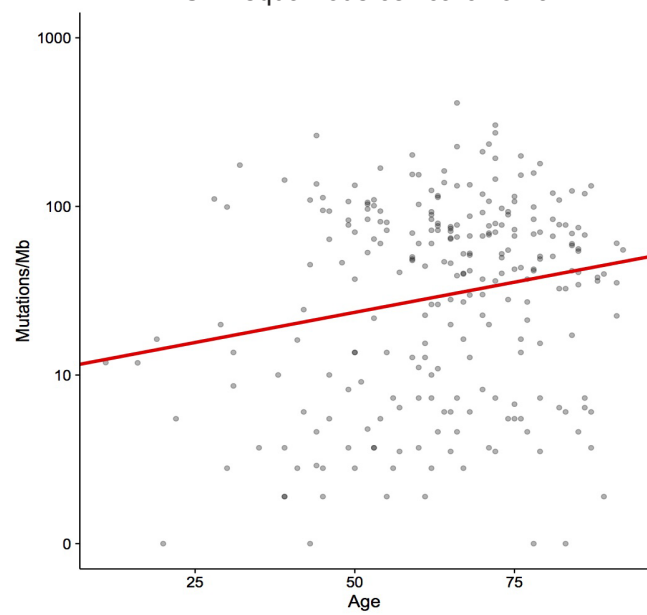

Colon adenocarcinoma

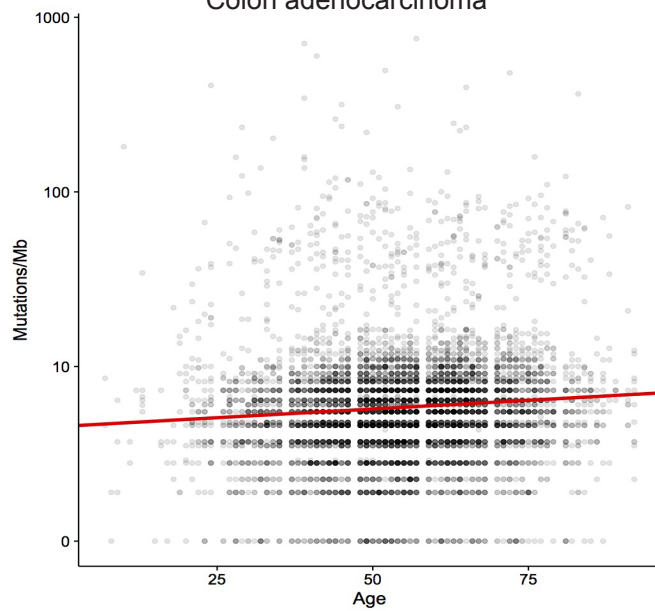

Supplement: Supplementary file 4 — TMB by age in select disease types (pdf). TMB versus age is plotted for select disease types, lung adenocarcinoma, skin squamous cell carcinoma, and colon adenocarcinoma. The red line shows the fit from a linear regression model for that disease type. (PDF 940 kb) [file 13073_2017_424_MOESM4_ESM.pdf]

Number of specimens with mutation

MSH6

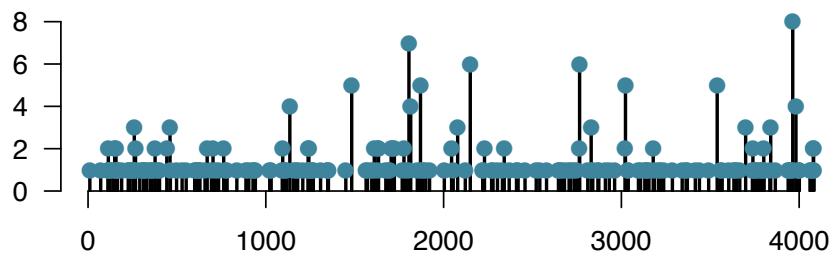

MLH1

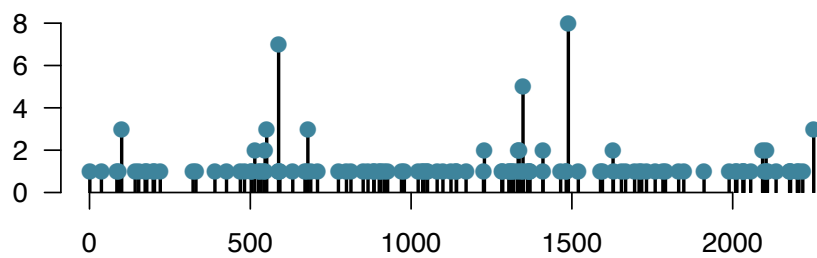

MSH2

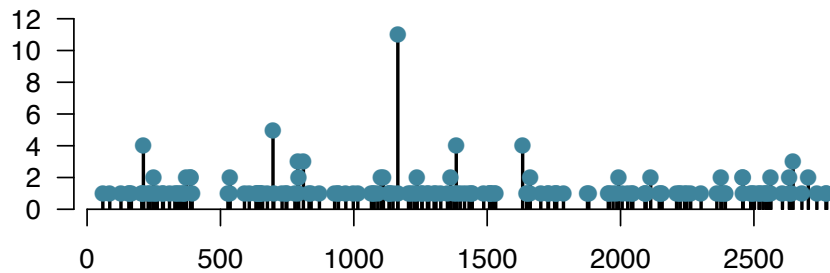

PMS2

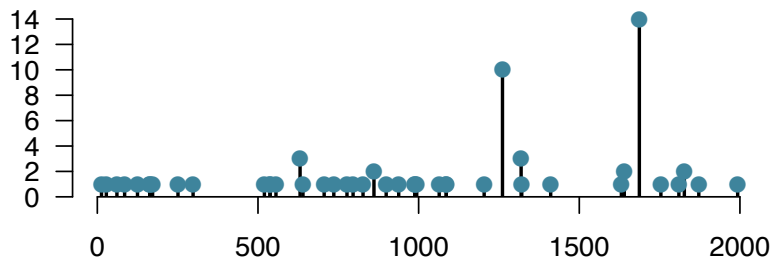

POLE

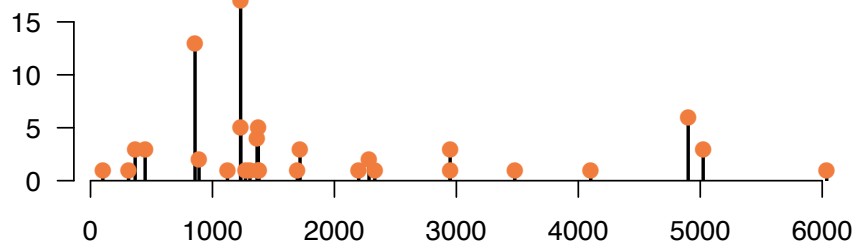

Supplement: Supplementary file 6 — Location of known or likely functional mutations in mismatch repair and POLE genes (pdf). For the genes MSH6, MLH1, MSH2, PMS2, and POLE, the count of mutations at each position in the transcript is plotted. (PDF 37 kb) [file 13073_2017_424_MOESM6_ESM.pdf]
